# Delirium in postoperative nonventilated intensive care patients: risk factors and outcomes

**DOI:** 10.1186/2110-5820-2-51

**Published:** 2012-12-31

**Authors:** Rodrigo Bernardo Serafim, Maximiliano F Dutra, Felipe Saddy, Bernardo Tura, Jose Eduardo Couto de Castro, Luciana C Villarinho, Maria da Gloria Santos, Fernando Augusto Bozza, José Rodolfo Rocco

**Affiliations:** 1 Ventilatory Care Unit, Copa D’Or Hospital, Rio de Janeiro, Brazil; 2 D’Or Institute of Research and Education, Rio de Janeiro, Brazil; 3 Department of Internal Medicine and Post-graduated Program, Hospital Universitário Clementino Fraga Filho, Federal University of Rio de Janeiro, Rio de Janeiro, Brazil; 4 Surgical Intensive Care Unit, Copa D’Or Hospital, Rio de Janeiro, Brazil; 5 ICU, Instituto de Pesquisa Clínica Evandro Chagas, Fundação Oswaldo Cruz, Rio de Janeiro, Brazil

**Keywords:** Delirium, Postoperative, Surgery, Confusion assessment method

## Abstract

**Background:**

Delirium features can vary greatly depending on the postoperative population studied; however, most studies focus only on high-risk patients. Describing the impact of delirium and risk factors in mixed populations can help in the development of preventive actions.

**Methods:**

The occurrence of delirium was evaluated prospectively in 465 consecutive nonventilated postoperative patients admitted to a surgical intensive care unit (SICU) using the confusion assessment method (CAM). Patients with and without delirium were compared. A multiple logistic regression was performed to identify the main risk factors for delirium in the first 24 h of admission to the SICU and the main predictors of outcomes.

**Results:**

Delirium was diagnosed in 43 (9.2%) individuals and was more frequent on the second and third days of admission. The presence of delirium resulted in longer lengths of SICU and hospital stays [6 days (3–13) vs. 2 days (1–3), *p* < 0.001 and 26 days (12–39) vs. 6 days (3–13), *p* <0.001, respectively], as well as higher hospital and SICU mortality rates [16.3% vs. 4.0%, *p* = 0.004 and 6.5% vs. 1.7%, *p* = 0.042, respectively]. The risk factors for delirium were age (odds ratio (OR), 1.04 [1.02-1.07]), Acute Physiologic Score (APS; OR, 1.11 [1.04-1.2]), emergency surgery (OR, 8.05 [3.58-18.06]), the use of benzodiazepines (OR, 2.28 [1.04-5.00]), and trauma (OR, 6.16 [4.1-6.5]).

**Conclusions:**

Delirium negatively impacts postoperative nonventilated patients. Risk factors can be used to detect high-risk patients in a mixed population of SICU patients.

## Background

Delirium is a frequent and severe complication in critical care units and is associated with worse short- and long-term outcomes [1,2]. Delirium has been associated with an increased length of stay, poor functional recovery, higher mortality, and greater cost [2-6]. Although the diagnosis of delirium has received increased attention in the past few years, recent studies have shown that it is still an underdiagnosed condition and that modifiable risk factors related to its occurrence are frequently neglected [7,8].

Patient safety advocates have highlighted the prevention of delirium in the reduction of postoperative complications [9]. A high incidence of delirium has been described in mechanically ventilated patients and after hip fracture surgery and cardiac surgery [10,11]; however, the incidence can vary widely with the type of surgery, the underlying medical conditions, and the criteria used for diagnosis. Even though most studies have been focused on a specific population, many surgical intensive care unit (SICU) patients are not mechanically ventilated or even suffer a high-risk surgery. Because the risk factors for the development of delirium include a complex relationship between predisposing and precipitating factors [11-13], models that help to identify risk factors for stratification allow the identification of high-risk groups and provide the basis for specific prevention programs, particularly in the postoperative setting.

In this prospective study, we described the incidence of delirium using an algorithm based on the Confusion Assessment Method (CAM) [14], calculated using the association of delirium with mortality and length of stay, and assessed risk factors related to the development of delirium, particularly in a mixed, low-risk population, who is frequently neglected in SICU.

## Methods

### Patients and setting

The setting was a 12-bed SICU at a tertiary hospital in Rio de Janeiro, Brazil. After approval from the ethics committee, informed consent was obtained from the patients or their representatives before enrollment.

### Selection and description of participants

A total of 824 consecutive subjects admitted to the SICU between November 2005 and July 2006 were screened for inclusion in the study. Patients were included if they had an adequate level of consciousness, as determined by a Richmond Agitation-Sedation Scale (RASS) score greater than −3. Patients were excluded if one of the following factors were present: mechanical ventilation, pregnancy, age less than 18 years, inability to verbalize, considerable hearing or visual impairment, or lack of informed consent.

### Sedation status and CAM application

The RASS was used to assess sedation status and is a ten-point rating scale with four levels for agitation, five levels for sedation, and one level for calm, awake patients [15,16]. Two trained nurses applied the CAM for delirium evaluation. Briefly, CAM evaluates the four key delirium features: (1) acute onset and fluctuating course; (2) inattention; (3) disorganized thinking; and (4) altered level of consciousness. Delirium was considered present if features 1 and 2 were present in addition to either feature 3 or 4 [14]. The CAM tool was performed twice a day during the entire SICU stay, and evaluation started on the day shift after the patient’s arrival from surgery and continued until SICU discharge.

### Nurses training program for the CAM

The training program for nurses consisted of three steps. In step 1, information about delirium, including relevant literature, handouts, and CAM tools, was provided and a video about the detailed application of the CAM was shown. In step 2, one-to-one instruction at the patients’ bedside was performed. During this period, staff members were able to interact with the delirium experts regarding any issues with the screening. In step 3, patient evaluations that were completed by the delirium expert 1 week before were compared with the evaluations completed by the nurses. Diagnosis of delirium and the data collected by two nurses were compared at the same time of day for 1 week to confirm consistency between observers.

### Data collection and definitions

A standardized data entry form was created for the collection of the following: demographic data, major surgical diagnoses, comorbidities, laboratory results based on the computerized laboratory records necessary to calculate the APACHE II score, hemodynamic changes (fever, hypotension, hypoxemia, or need of oxygen therapy), and the use of benzodiazepines or opioids in the first 24 h of admission to the SICU. Data regarding comorbidities were collected with the anesthetic record and confirmed with family and patients. We considered comorbidities to be all medical conditions reported by the patients or their family during the pre-anesthetic interview. We considered emergency surgery when it was required in less than 24 h after surgeon evaluation. Acute Physiology and Chronic Health Evaluation (APACHE) II scores were calculated using data from the first 24 h of the SICU admission. The need to use hearing or ocular devices was investigated to be sure that the patient is able to understand the CAM interview.

### Statistical analysis

Continuous variables are summarized as medians and interquartile ranges (25th and 75th percentiles), and dichotomous categorical variables are presented as proportions of frequency. The result of the CAM was evaluated as a dichotomous variable (presence or absence of delirium). Nonparametric Mann–Whitney, Kruskal-Wallis, Spearman correlation coefficients, and chi-squared tests were performed to compare the association between the clinical and demographic variables and the development of delirium. A *p* value < 0.05 was considered significant. The odds ratio (OR) and 95% confidence interval (CI) were described. Intensive care unit and hospital mortality were of particular interest. We performed multivariate forward logistic regression relating the APACHE II score and the presence of delirium with mortality. Univariate and multivariate logistic regression were used to identify risk factors present in the first 24 h of SICU admission associated with the development of delirium. Variables yielding *p* < 0.2 by univariate analysis and those considered clinically relevant were entered in the multivariate analysis to estimate the independent association of each covariate with the dependent variable. Age, type of surgery, emergency surgery, APS, benzodiazepine use, hypoxemia, and hypotension were included in the model as risk factor for delirium. Hypotension and hypoxemia was positively related with delirium occurrence in univariate analysis, but after the multivariate analysis both variables did not promote a better model considering APS simultaneously. The Hosmer-Lemeshow goodness-of-fit test was used to verify the calibration of the risk-factor-to-delirium model developed using logistic regression.

SPSS®, version 10.0 (SPSS Inc., Chicago, IL) was used for statistical analysis.

## Results

**Figure 1:**
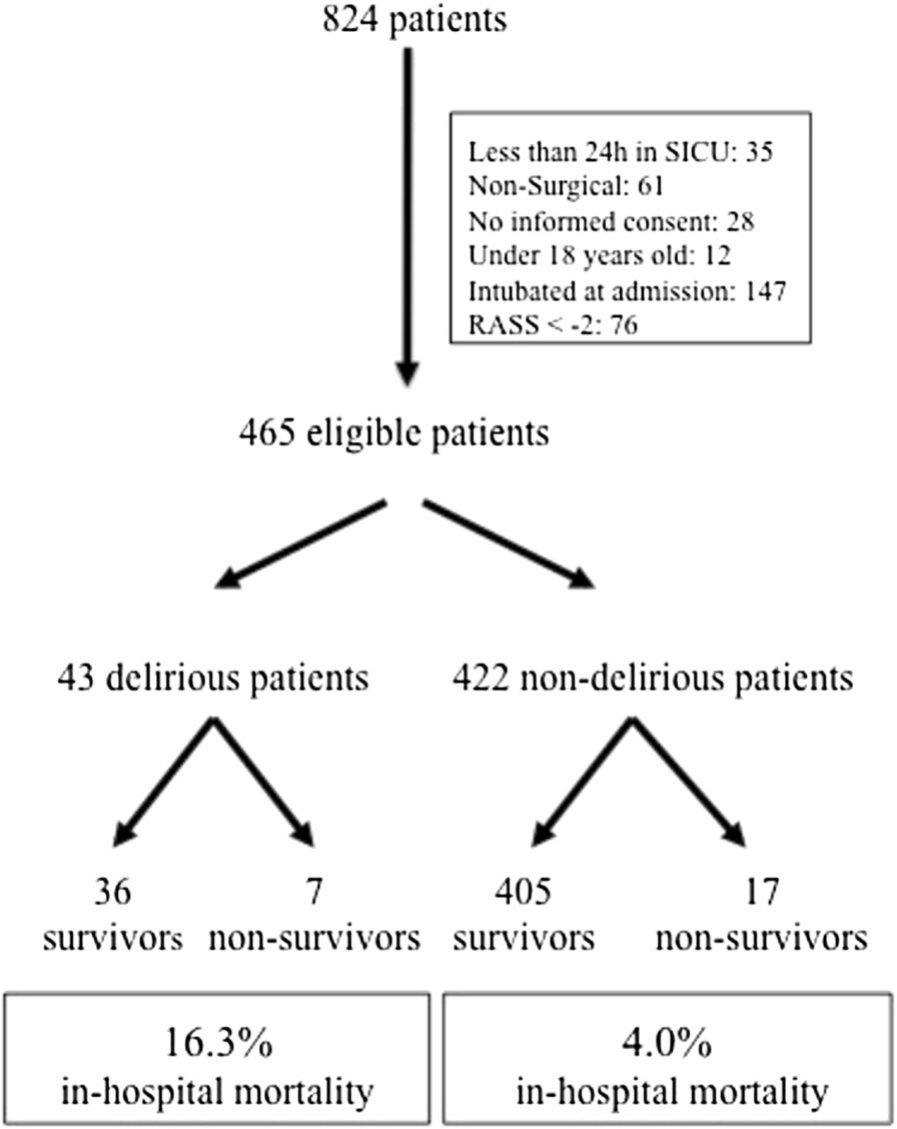
**Fluxogram of patient inclusion.** RASS, Richmond Agitation-Sedation Scale.

From the 824 patients screened during the study period, 465 were eligible for inclusion and were analyzed (Figure 1). The main demographic data are shown in Table 1.

**Table 1:** Clinical and surgical summary of patients with and without delirium

**Variable**	**All patients (n = 465)**	**Delirium (n = 43)**	**Non-delirium (n = 422)**
Gender (M:F)	1.1:1		
Age, yr (IQR)	60 (47–74)	73 (60–82)	59 (46–73) *
Apache II score (IQR)	10 (6–13)	13 (11–17)	10 (6–13) *
APS (IQR)	7 (3–10)	9 (6–11)	7 (2–10) *
**Patients comorbidities**			
Diabetes mellitus (%)	51 (11%)	5 (11%)	46 (9.9%)
Hypertension (%)	138 (29.7%)	12 (27%)	126 (27.1%)
Chronic pulmonary disease (%)	13 (2.8%)	0	13 (2.8%)
Cancer (%)	30 (6.45%)	3 (6.5%)	27 (5.8%)
Chronic renal disease (%)	6 (1.3%)	1 (2%)	5 (1.1%)
Dementia (%)	7 (1.5%)	5 (11%)	2 (0.4%) *
**Site of surgery**			
Abdominal (%)	192 (41.3%)	15 (34.0%)	177 (42.0%)
Orthopedic (%)	96 (20.6%)	9 (20.9%)	87 (20.6%)
Head and neck (%)	38 (8.2%)	5 (11.6%)	33 (7.8%)
Urologic (%)	35 (7.5%)	2 (4.8%)	33 (7.8%)
Vascular (%)	35 (7.5%)	2 (4.8%)	33 (7.8%)
Thoracic (%)	24 (5.2%)	1 (2.4%)	23 (5.6%)
Cardiac (%)	19 (4.1%)	2 (4.8%)	17 (4%)
Trauma (%)	18 (3.9%)	7 (16.3%)	11 (2.6%) *
Gynecological (%)	8 (1.7%)	0	8 (1.9%)
Emergency surgery (%)	127 (27.3%)	34 (78%)	93 (22%) *
**Outcomes**			
In-hospital length of stay (IQR)	7 (4–14)	26 (12–39)	6 (3–13) *
In-hospital mortality (%)	24 (5.2%)	7 (16.3%)	17 (4.0%) *
SICU length of stay (IQR)	2 (1–4)	6 (3–13)	2 (1–3) *
SICU mortality (%)	9 (1.7)	3 (6.5%)	6 (1.7%) *

*M/F*, male/female; *APS*, Acute Physiologic Score; *IQR*, interquartile range. *p < 0.05.

**Figure 2:**
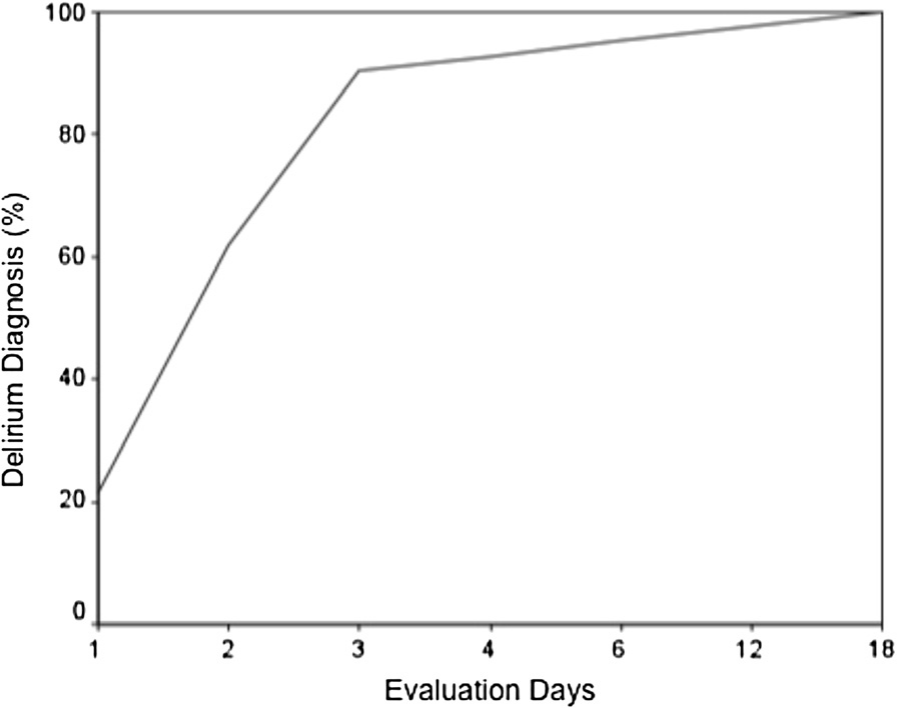
**Cumulative number of days until delirium diagnosis.** This figure shows the percentage of cumulative delirium. Approximately 91% of delirium cases occurred within the first 3 days after SICU admission. There were 9 (21.4%) cases in the first 24 h, 17 (40.5%) on the second day, and 12 (28.6%) on the third day after admission.

Delirium was diagnosed in 43 (9.2%) patients, and approximately 91% of these cases occurred within the first 3 days of SICU admission. There were 9 (21.4%) new cases in the first 24 h, 17 (40.5%) on the second day, and 12 (28.6%) on the third day after admission (Figure 2). There was no difference in the frequency of the diagnosis of delirium when the morning and afternoon evaluations were compared (52.9% vs. 47.1%, *p* = 0.06).

Compared with patients without delirium, patients with delirium had a higher median age (73 vs. 59 years, *p* < 0.001) and a higher median Apache II score (13 vs. 10; *p* < 0.001). Delirium was more frequent after emergency surgeries (78% vs. 22%; *p* <0.001), and there was no relationship between the site of surgery and the presence of delirium. Dementia was the only comorbidity that was statistically associated with delirium, but it was only present in a small number of patients (Table 1).

### Delirium outcomes

The median lengths of SICU and hospital stays were significantly increased in patients who developed delirium. Patients with delirium had higher hospital and SICU mortality rates compared with patients without delirium (Table 1).

**Figure 3:**
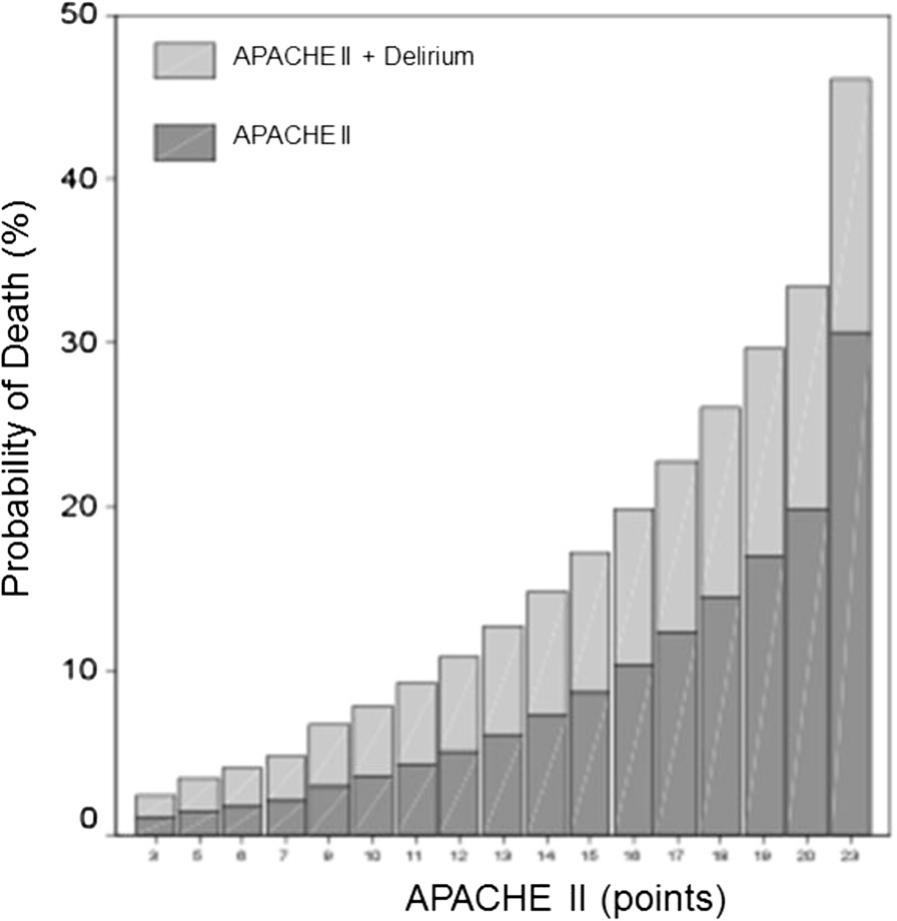
**Influence of delirium on the probability of death related to the APACHE II score.** This figure describes the increase in the probability of hospital mortality according to each point of APACHE II score. APACHE, Acute Physiology and Chronic Health Evaluation II.

The relative risk of hospital mortality associated with delirium and APACHE II scores (each point) was 1.367 (1.135-1.999) and 1.194 (1.097-1.299), respectively. Moreover, as shown in Figure 3, the occurrence of delirium impacted the mortality related to APACHE II score by increasing the probability of death.

### Delirium risk factors

The risk factors related to delirium were as follows: age (years) (OR, 1.04 [1.02-1.07]), APS (each points) (OR, 1.11 [1.04-1.2]), emergency surgery (8.05 [3.58-18.06]), the use of benzodiazepines in the first 24 h after admission (OR, 2.28 [1.04-5]), and trauma (OR, 6.16 [4.1-6.5]; Table 2).

**Table 2:** Risk factors for delirium

**Variable**	**OR (CI)**
Age (each year)	1.04 (1.02–1.07)
APS score (each point)	1.11 (1.04–1.2)
Benzodiazepine use (Y/N)	2.28 (1.04–5)
Emergency surgery (Y/N)	8.05 (3.58–18.06)
Trauma patients (Y/N)	6.16 (4.1–6.5)

*APS*, Acute Physiologic Score; *Y/N*, yes/no.

The ROC curve was used to evaluate the accuracy of these risk factors. The ROC curves for age and APS were produced separately, because they represented two classic risk factors for the occurrence of delirium. However, in this case, the curves showed areas under the curve for APS and age of 0.638 ± 0.44 (0.551–0.724) and 0.673 ± 0.045 (0.585–0.761), respectively. When all risk factors were combined to predict delirium, the area under the curve was 0.89 ± 0.021 (0.85–0.931). The statistics of the goodness-of-fit test showed a satisfactory calibration (C = 3.47; *p* = 0.901).

## Discussion

This prospective cohort study clearly demonstrates the association of delirium with the increase in length of stay and mortality in a nonventilated elective and emergency SICU population. Additionally, we identified the main risk factors associated with delirium in this population.

The CAM tool was preferred, because it was already validated in Portuguese [17] and it is seen to be noninferior for the diagnosis of delirium compared with other tools, including the CAM-ICU in nonventilated patients [18].

In this study, the delirium incidence was low (9.2%), although the population studied was predominantly elderly, and aging groups are clearly at risk for the development of delirium [19,20]. Low severity scores and short lengths of stay in our population most likely contributed to a reduced delirium incidence.

Most delirium episodes occurred in the first 3 days of admission (91%), which is similar to the findings in mechanically ventilated medical ICU patients [1]. Interestingly, in our population, delirium occurred most frequently between the second and third day, but not on the first day, which could be expected given the chronological proximity of the surgical and anesthetic procedures. However, this timeline for development of delirium is consistent with the peak levels of inflammatory mediators in the postoperative period, such as interleukin-6 (which peaks after 24 h) and C-reactive protein (which peaks after 48 h) [21]. It also has been demonstrated that patients with the greatest increases in inflammatory mediators are more likely to experience postoperative delirium [22].

There was no significant relationship between the site of surgery and the occurrence of delirium. This finding may be explained by the small sample sizes of the different types of surgery, which may prevent a more accurate comparison. However, previous studies demonstrated an association between large cardiovascular and orthopedic procedures and the development of delirium [11,23].

We observed that the development of delirium at any time during the postoperative ICU hospital stay impairs the prognosis of SICU patients. Those who developed delirium during the SICU stay had a significantly higher mortality in both the SICU and the hospital compared to patients without delirium. This finding is similar to that described in both clinical [1] and surgical [24] patients. The occurrence of delirium increased the probability of death related to the APACHE II score. This impact was higher in terms of absolute numbers in patients with higher APACHE II scores; however, the lowest scores had the greatest proportional increase in the probability of death, as shown in Figure 3.

Among the risk factors analyzed for the occurrence of delirium, age (each year) and APS (each point) were the most important, as shown by the highest proportional relative risks. Others factors had a large variation in the CI, which may be due to the small sample size and should be interpreted with caution.

The management of delirium in the SICU includes early detection and enforcement of nonpharmacological control [24-26]. One recent study showed a benefit of using perioperative antipsychotics in preventing delirium; however, there is concern about the increase in the incidence of hypoactive delirium as a result of this practice [27].

Because the development of delirium has a major impact in the SICU population, risk factors and time to delirium occurrence described in the present study can help in the development of preventive actions against the development of delirium, enabling better resource allocation, providing useful family information and determining more accurate evaluation of outcomes in postoperative patients.

This study presents some limitations, including the fact that this was a single-center study. The intraoperative data were not collected systematically, but data from the literature regarding anesthesia are conflicting, and it is unclear whether the type of anesthesia affects the development of delirium [28]. Patients with RASS −3 were excluded contributing to the lower incidence of delirium in this population. There have been changes in medical and surgical care since data collection, but recent surveys show that delirium recognition and preventive practices in SICU patients must still be improved [7,8]. Patients were not followed up after hospital discharge to determine subsequent mortality or cognitive dysfunction.

## Conclusions

The features of delirium can vary depending on the population studied. The results of this prospective study provide data about the impact of delirium and its risk factors and outcomes in a general nonventilated postoperative low severity population. Delirium was associated with a significant increase in hospital mortality, and it had a negative impact even in patients with lower APACHE II scores. We also found that age, APS score, emergency surgery, trauma, and the use of benzodiazepines in the first 24 h of admission were risk factors associated with delirium in this population. These findings could be useful in the development of prevention programs in the SICU.

## Competing interests

The authors declare that they have no competing interests.

## Authors’ contributions

SRB and DM were responsible for the data input. VLC and SMG were responsible for the data collection. BF, CJEC, RJR, and SF composed the manuscript. BF and RJR provided editorial assistance. TB provided the statistical analysis. All authors read and approved the final manuscript.
